# A transcriptomic computational analysis of mastic oil-treated Lewis lung carcinomas reveals molecular mechanisms targeting tumor cell growth and survival

**DOI:** 10.1186/1755-8794-2-68

**Published:** 2009-12-15

**Authors:** Panagiotis Moulos, Olga Papadodima, Aristotelis Chatziioannou, Heleni Loutrari, Charis Roussos, Fragiskos N Kolisis

**Affiliations:** 1 Metabolic Engineering and Bioinformatics Group, Institute of Biological Research and Biotechnology, National Hellenic Research Foundation, 48 Vasileos Constantinou ave. 11635, Athens, Greece; 2 "G.P. Livanos and M. Simou Laboratories", Evangelismos Hospital, Department of Critical Care and Pulmonary Services, School of Medicine, University of Athens, 2 Ploutarchou st., 10676, Athens, Greece

## Abstract

**Background:**

Mastic oil from *Pistacia lentiscus *variation *chia*, a blend of bioactive terpenes with recognized medicinal properties, has been recently shown to exert anti-tumor growth activity through inhibition of cancer cell proliferation, survival, angiogenesis and inflammatory response. However, no studies have addressed its mechanisms of action at genome-wide gene expression level.

**Methods:**

To investigate molecular mechanisms triggered by mastic oil, Lewis Lung Carcinoma cells were treated with mastic oil or DMSO and RNA was collected at five distinct time points (3-48 h). Microarray expression profiling was performed using Illumina mouse-6 v1 beadchips, followed by computational analysis. For a number of selected genes, RT-PCR validation was performed in LLC cells as well as in three human cancer cell lines of different origin (A549, HCT116, K562). PTEN specific inhibition by a bisperovanadium compound was applied to validate its contribution to mastic oil-mediated anti-tumor growth effects.

**Results:**

In this work we demonstrated that exposure of Lewis lung carcinomas to mastic oil caused a time-dependent alteration in the expression of 925 genes. GO analysis associated expression profiles with several biological processes and functions. Among them, modifications on cell cycle/proliferation, survival and NF-*κ*B cascade in conjunction with concomitant regulation of genes encoding for PTEN, E2F7, HMOX1 (up-regulation) and NOD1 (down-regulation) indicated some important mechanistic links underlying the anti-proliferative, pro-apoptotic and anti-inflammatory effects of mastic oil. The expression profiles of *Hmox1*, *Pten *and *E2f7 *genes were similarly altered by mastic oil in the majority of test cancer cell lines. Inhibition of PTEN partially reversed mastic oil effects on tumor cell growth, indicating a multi-target mechanism of action. Finally, k-means clustering, organized the significant gene list in eight clusters demonstrating a similar expression profile. Promoter analysis in a representative cluster revealed shared putative *cis*-elements suggesting a common regulatory transcription mechanism.

**Conclusions:**

Present results provide novel evidence on the molecular basis of tumor growth inhibition mediated by mastic oil and set a rational basis for application of genomics and bioinformatic methodologies in the screening of natural compounds with potential cancer chemopreventive activities.

## Background

Lung cancer is the leading cause of cancer deaths in the US among both men and women [1]. Nowadays the search for new chemopreventive and chemotherapeutic agents to treat malignancies, especially the most mortal types characterized by rapid metastasis and frequent resistance to current chemotherapy/radiotherapy regimens has recently increased and the interest is mainly focused on natural compounds with low toxicity [2]. A large body of pre-clinical, clinical and epidemiological studies support that many phytochemicals, i.e. bioactive compounds isolated from plants, can delay tumor progression and metastasis [3,4]. While most of the available evidence refers to isolated substances, recent data support that natural combinations of phytochemicals in extracts often possess enhanced reactivity due to their additive and/or synergistic interactions [5]. Plant essential oils containing a wide spectrum of compounds seem to be promising in this respect. Mastic oil, the essential oil of mastic gum, a natural resin obtained from *Pistacia lentiscus *variation *chia *has been extensively used in the Mediterranean and Middle Eastern countries as food/beverages flavouring additive and traditional medicine since antiquity without any reported toxicity. Chemical composition analysis of mastic oil revealed that it is a complex mixture of volatile compounds, mainly terpenes, with established beneficial biological properties [6,7]. Although these compounds have been shown to inhibit a variety of tumor-promoting cellular pathways in cancer cells, their precise mechanism(s) of action is still uncertain. It appears that plant-derived terpenes act primarily as inhibitors of the mevalonate pathway which regulates the biosynthesis of specific isoprenoids that are indispensable to the post-translational modification of small GTPase [8,9]. Regarding the health beneficial properties of mastic oil, it has been proved to act as antimicrobial [6,7], anti-inflammatory [10] and anti-atherogenic [11] agent without substantial side effects in humans and animals [10,12].

Furthermore, recent studies have revealed that mastic extracts can also exert anti-tumor growth activities against several cancer types (leukemia, prostate, colon, lung and melanoma cancer cells) through mechanisms involving inhibition of tumor cell proliferation and survival, restriction of angiogenesis and modulation of pro-tumor inflammatory response [13-17]. In addition, mastic oil treatment has been shown to target the expression and function of key signaling and transcription regulators implicated in malignant phenotype like Ras/RhoA GTPases and NF-*κ*B [14,17].

Despite the great number of reports analyzing the action mechanisms of plant derived compounds, studies focusing on their effects at the gene expression level are very limited. In this work, by combining sensitive high-throughput transcriptomic technology and bioinformatics on Lewis lung adenocarcinoma (LLC) cells, a susceptible to mastic oil cancer cell line [17], we were able to evaluate for the first time the differential expression of tumor genes in a genome-wide scale and identify target pathways modified in response to mastic oil treatment. Furthermore, by analyzing the expression of selected target genes in three additional human cancer cell lines we confirmed that the anti-tumor effects of mastic oil were more general. Our results might help to delineate the molecular basis of mastic oil chemopreventive/chemotherapeutic actions.

## Methods

### Cell culture and treatment

LLC and K562 cells were cultured in DMEM and RPMI 1640, respectively. Media were supplemented with 10% FCS, L-glutamine and antibiotics. HCT116 cells were maintained in DMEM supplemented with 10% FCS, nonessential amino acids and antibiotics. A549 cells were maintained in F19-K supplemented with 10% FCS and antibiotics. All cell lines were originally obtained from American Type Culture Collection, Manassas, VA and all media and supplements were from Gibco, Grand Island, NY. For RNA isolation, LLC cells were plated in 6-well plates at 2.3 × 10^5 ^cells/well, K562 in 24-well plates at 3 × 10^5 ^cells/well, HCT116 in 12-well plates at 4 × 10^5 ^cells/well and A549 were plated in 12-well plates at 1.5 × 10^5 ^cells/well. 24 h later cells were treated with mastic oil (0.01% for LLC and 0.02% v/v for the other cell lines) or 0.1% DMSO vehicle for the indicated periods of time (3 to 48 h).

### RNA isolation

RNA was isolated with the Trizol Plus kit (Invitrogen, Carlsbad, CA, USA) according to the manufacturer instructions. The quantification and quality analysis of RNA was performed on a Bioanalyzer 2100 (Agilent, Santa Clara, California).

### Quantitative RT-PCR

Reverse Transcription and Real-Time PCR was performed with the TaqMan RNA-to-C_T _1-Step Kit. In the case of mouse genes TaqMan primers and probes were from Applied Biosystems, Foster City, California, while for human genes primers and probes were from Integrated DNA Technologies, Germany. The reactions were performed with 0.5 μg of RNA as template, according to the manufacturer's instructions. Amplifications were performed on a 7500 Real-Time PCR System (Applied Biosystems Foster City, California) as follows: reverse transcription step at 48°C for 15 min, followed by incubation at 95°C for 10 min and 40 cycles of 95°C for 15 s and 60°C for 1 min. *Gapdh *gene was used as the reference gene for all normalizations. The sequence of primers and probes for mouse genes is as follows: *Hmox1*: Forward primer: 5'-GGTTGTAAGCATCCATGTTGACTGA-3', Reverse primer: 5'-ACAGAAATGTCTGGAAACGGATATCAA-3', Probe: 5'-CCAGTGCCATGGCCAC-3', *Nod1*: Forward primer: 5'-GAGCTGCACTCAGACTTCGA-3', Reverse primer: 5'-CCAGAGGGTGAGCCGG-3', Probe: 5'-CCGCGTGCCGGATAG-3', *Pten*: Forward primer: 5'-AGATATTCTGACACCACTGACTCTGA-3', Reverse primer: 5'-CAGACTTTTGTAATTTGTGAATGCTGATCTT-3', Probe: 5'-TCCAGAGAATGAACCTTTTG-3', *E2f7*: Forward primer: 5'-CTCACACGGCGTCATCCA-3', Reverse primer: GCTCTGCCTTTACCATCGATACC-3', Probe: 5'-CCTGTTACGTGAGACATC-3', *Gapdh*: Forward primer: 5'-GTGTCCGTCGTGGATCTGA-3', Reverse primer: 5'-GCTTCACCACCTTCTTGATGTCAT-3', Probe: 5'-CTTGGCAGGTTTCTCC-3'. The sequence of primers and probes for human genes is as follows: *Hmox1*: Forward primer: 5'-CCCCAACGAAAAGCACAT-3', Reverse primer: 5'-TGGAGGTTTGAGACAGCT-3', Probe: 5'-CCCCTCTGAAGTTTAGGCCATTGC-3', *Nod1*: Forward primer: 5'-TCTTCCTCTACTTGCTCCAG-3', Reverse primer: 5'-GTTGACCACGACTTTGCT-3', Probe: 5'-CTTCTCCCCTTCCCTGCTCACT-3', *Pten*: Forward primer: 5'-CAATGTTCAGTGGCGGAA-3', Reverse primer: 5'-TCGTGTGGGTCCTGAATT-3', Probe: 5'-TCACCTTTAGCTGGCAGACCACAA-3', *E2f7*: Forward primer: 5'-TTCTACTCTTGGTGCTCTCC-3', Reverse primer: 5'-AGCTGGGCTATTGATCCA-3', Probe: 5'-ACCTGTGAATTTCAGCTTGCCTGG-3', *Gapdh*: Forward primer: 5'-TGACCTGCCGTCTAGAAA-3', Reverse primer: 5'-GTGTCGCTGTTGAAGTCA-3', Probe: 5'-AGTGTAGCCCAGGATGCCCTT-3'. Each reaction was analyzed in triplicates and data analysis was performed according to the  method.

### Cell growth assay

K562 cells were plated in 96-well plates (4 × 10^3 ^cells/well) and 24 h later were treated with fresh media containing mastic oil (0.007% v/v), potassium bisperoxo (1,10-phenanthroline) oxovanadate (bpV(phen)) (1.0 μM) (Calbiochem, Merck, Darmstadt, Germany), mastic oil (0.007% v/v) together with bpV(phen) (0.5 and 1.0 μM), or DMSO (0.1%). The above concentrations of bpV(phen) were selected on the basis of earlier work showing that bpV(phen) is a specific inhibitor of PTEN at a concentration of 1 μM or lower [18]. After 24 h of incubation, cell numbers were determined using CellTiter 96 AQ_ueous _One Solution Cell Proliferation Assay (Promega, Madison, USA) according to manufacturers' instructions.

### Microarray hybridizations and data analysis

Synthesis of cDNA and biotinylated cRNA was performed with the Illumina TotalPrep RNA Amplification Kit (Illumina, San Diego, California) using 500 ng of total RNA. Hybridization was performed in duplicates onto Illumina Mouse-6 v1 Expression BeadChips according to manufacturer's instructions. The expression dataset, produced from the hybridization onto Illumina Mouse-6 v1 Expression BeadChips, was corrected for background noise using Illumina's negative controls, representing an estimation of both image background and signals derived from non-specific binding or cross-hybridizations. Data were normalized using the Rank Invariant normalization algorithm implemented on Illumina BeadStudio software, using all probes on the array for the extraction of the rank invariant set of genes. After normalization, data were filtered using Illumina's "detection score" which represents the probability that each probe's bead signal distribution does not overlap with the bead distribution of the negative control samples. A gene was considered "present" (P) if the detection was above 0.99. Genes scoring below 0.98 were considered as "absent" (A) while genes scoring between 0.98 and 0.99 as "marginally present" (M). Genes absent in all beadchips (20 in total) or present only in one beadchip were excluded from further analysis. Data were also normalized with Quantile normalization [19], with data filtering applied before normalization to alleviate it from the impact of systematic measurement errors to produce a second dataset of gene expression values.

To deal with the problem of negative signal values or values below 1 caused by the background subtraction step, all the remaining values below 1 were transformed to missing values. Subsequently, the experimental time points were divided into 10 subcategories (5 pairs of each time point's mastic oil treatment and its corresponding control), each consisting of duplicate expression values for each gene. For each subcategory, if the expression value for a gene was missing in both duplicate measurements, imputation was performed by replacing the missing value with 1. If the expression value existed in one of the duplicates, then the existing value was used for the imputation of the other duplicate measurement according to the following strategy: expression values from the beadchip with the missing gene were divided in 20 physical intensity bins. The weighted mean of the 30 nearest neighbours (in terms of absolute distance) of the non-missing value from the bin where this value belonged to, was used to impute the missing one. After the imputation step, genes were further characterized as "weak" (W) if their expression value fell below the 5^th ^percentile of all expression values from the respective beadchip, "intermediate" (I) if the expression value was between the 5^th ^and 20^th ^percentile and "strong" (S) if it laid above the 20^th ^percentile.

### Statistical analysis

Differentially Expressed (DE) genes in at least one among all the experimental time points were identified by performing 1-way ANOVA on log_2 _transformed fold changes for each time-point. Fold change values were calculated for each gene as the ratio of the mastic oil to vehicle treatments. The resulting gene list was obtained by setting the p-value threshold to 0.05 and by removing genes that presented a fold change below |0.2| (in log_2 _scale), in all time points. The described procedures were applied to both Rank Invariant and Quantile normalized data and the final gene list was obtained by the intersection of the lists, derived by the two normalizations. Noisy genes that remained after the initial filtering steps and characterized as WA, IA or WM in all time points, were removed. For the calculation of each gene's final p-value, an approach described before in [20] was used: as the dataset was normalized with two independent normalization methods, two different datasets having different value sets for each gene were produced. These normalized datasets were subjected to statistical testing, producing two different p-value distributions. The p-value for each gene equals the product of the two separate p-values, produced by the statistical test for each normalization algorithm, due to their independent nature. Subsequently, the new p-value distribution was used to estimate the FDR levels, based on the method described in [21]. The final gene list corresponds to an FDR < 0.05.

### Ontological analysis

The final set of genes was analyzed according to the corresponding Gene Ontology Terms (GOTs) to these genes, for the identification of groups of genes referring to the same biological process or cellular biochemical pathway, using an algorithm presented in [20] currently assembled in a software package [22]. To further examine the significance of the extent of kinship for the resulting population of enriched GOTs, related to the categories 'Molecular Function' (MF) and 'Biological Process' (BP), a graph theoretic pairwise absolute distance metric ([23] and references therein), of the relevant GO tree was formulated and statistically tested:

where *n*_*c *_denotes the number of GOTs in either category (MF or BP) and  denotes the absolute distance between nodes *i *and *j *in the GO Tree Undirected Acyclic Graph (UAG). *D*_*c *_represents the average of all the pairwise absolute distances between two GOTs, in the GO UAG calculated for both MFs and BPs. The GO Directed Acyclic Graph (DAG) was retrieved using tools from MATLAB's 7.4 (R2007a) Bioinformatics Toolbox and converted to an Undirected Acyclic Graph (UAG) by transforming its adjacency matrix in order to measure absolute distances between DAG nodes. The aforementioned metric was applied for the case of MF and BP to both GOT populations (significant GO set and whole chip GO set).

The statistical validity of *D*_*c*_, was assessed through resampling (1000 times) the same number of MF GOTs and BP GOTs as those in Table 1, and from the respective whole chip GO sets (background), resulting in two background (MFs and BPs) bootstrap distributions of *D*_*c*_'s. Statistical significance was assessed using the bootstrap p-value:

**Table 1:** GO-analysis of gene expression alterations over time

GO term annotation	Category	HT p-value	Enrichment
kinase binding	MF	0.0000013	4/11
histone acetyltransferase activity	MF	0.0000017	6/30
glutathione transferase activity	MF	0.0000064	6/36
BMP signalling pathway	BP	0.0000081	4/15
Oxidoreductase activity	MF	0.0000118	41/1019
DNA replication initiation	BP	0.0000295	4/19
Acyltransferase activity	MF	0.0000537	14/224
magnesium ion binding	MF	0.0000696	25/554
***negative regulation of cell proliferation***	BP	0.0002658	10/152
***cell cycle***	BP	0.0004519	27/694
lipoprotein metabolic process	BP	0,0004900	3/19
transcription factor binding	MF	0.0006180	9/142
lipid transport	BP	0.0007125	7/96
glutathione metabolic process	BP	0.0010504	3/23
fatty acid metabolic process	BP	0.0011307	7/103
cytoskeletal protein binding	MF	0.0012260	6/81
protein amino acid dephosphorylation	BP	0.0012779	12/241
regulation of translational initiation	BP	0.0016908	3/26
***positive regulation of I-κB kinase/NF-κB ****cascade*	BP	0.0019357	5/65
Phosphoprotein phosphatase activity	MF	0.0021059	12/255
***induction of apoptosis***	BP	0.0022511	8/141
cell division	BP	0.0023318	14/321
positive regulation of transcription from RNA polymerase II promoter	BP	0.0024536	11/229
transcription regulator activity	MF	0.0026217	14/325
nucleotide metabolic process	BP	0.0029513	4/49
electron transport	BP	0.0031794	23/645
transcription coactivator activity	MF	0.0032915	7/122
ubiquitin-protein ligase activity	MF	0.0034684	11/239
negative regulation of transcription	BP	0.0035622	8/151

The list of 925 significantly altered genes was submitted to GO analysis elucidating over-represented GO terms. HT p-value represents the hypergeometric test p-value score for each GO term. Enrichment represents the ratio of the number of times a GO term occurs in the significant gene list to the number of times this GO term exists in the list of the entire array. Selected categories for further analysis are shown in bold, italics.

where *m *is the number of resampling iterations.  is a modification of the estimation of the *Achieved Significance Level for bootstrap*, [24].

### Cluster analysis

The final gene list was subjected to k-means clustering, using Pearson correlation distance to identify groups of genes presenting similar expression profiles. To determine the optimal number of clusters, k-means algorithm was executed with k ranging from 2 to 30 and the Gap statistic [25] was used to estimate the optimal number k. This procedure was repeated 100 times so as to obtain a distribution of k_i _parameters, i = 1...100. The optimal cluster number was the value with the highest appearance frequency in the k_i _distribution and was found to be 8. All calculations apart from background correction and Rank Invariant normalization were performed using MATLAB 7.4 (R2007a).

### Promoter sequence retrieval and analysis

We downloaded promoter sequences from -500 to +100, relative to transcription start site, for each gene in cluster 2 for mouse and human from Cold Spring Harbor Laboratory Mammalian Promoter Database [26]. In the cases that alternative promoters were given for the same gene, we selected the one defined as the "best" [26]. For promoters that we could not detect in this database, we additionally searched the ElDorado database [27]. In the case of genes with multiple promoters supported by different transcripts, we selected the one corresponding to the Reference Sequence of NCBI. Out of 78 cluster 2 genes, we were able to obtain promoter sequences for 78 and 69 genes for mouse and human, respectively. To analyze each promoter set for common TF binding sites, we used the MatInspector software [28]. The parameters used were as follows: Library version: Matrix Library 8.0, Matrix group: Vertebrates, Transcription Factor sites common to: 65% of input sequences, Core similarity: 0.75, Matrix similarity: Optimized and p-value cut-off was set at 0.01. Among the identified TF sites only those that were present in both species were considered.

## Results

### Statistically significant differentiated genes

To obtain the gene expression profile after treatment with mastic oil for five distinct time points (3, 6, 12, 24 and 48 h), LLC cells were cultured with 0.01% v/v mastic oil or 0.1% DMSO, mRNA was isolated from and subjected to high-throughput gene expression profiling using high density oligonucleotide Illumina beadchips to analyze gene expression changes. The complete dataset has been deposited in NCBI's Gene Expression Omnibus (GEO) and is accessible through GEO Series accession number GSE15287. Proper pre-processing was applied to the expression values of the dataset in order to address the issues of noise and missing values. Subsequently, the generated dataset was normalized with two widely used microarray data normalization methods, as there exists no gold standard normalization method, with the scope to decrease the number of possible false positives during the statistical selection step.

**Figure 1:**
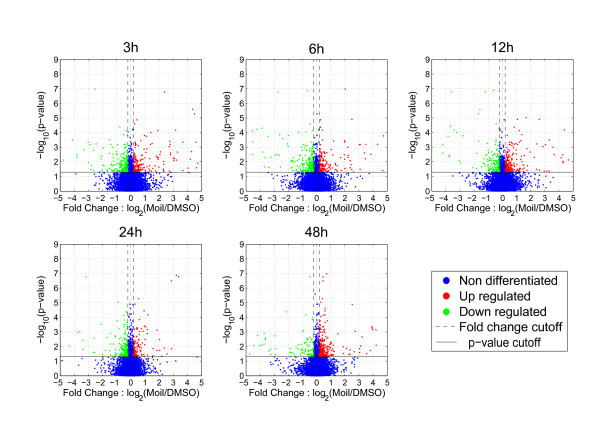
**Volcano plots of the gene list as yielded by ANOVA**. Each panel represents filtered and normalized data from each experimental time point (3, 6, 12, 24 and 48 h). The horizontal axes depict the fold change ratio between treatment with mastic oil and vehicle for each time point in log_2 _scale while the vertical axes represent statistical significance by depicting the -log_10_(p-value). Larger values in the vertical axes represent larger statistical significance. Differentially expressed genes (fold change>|0.2| in log_2 _scale, p-value < 0.05, FDR < 0.05) are shown in each panel where down-regulated genes are depicted by green dots and up-regulated genes by red dots. Non differentiated genes are shown as blue dots. Dashed lines indicate fold change thresholds and solid lines indicate the statistical significance (p-value) threshold. The p-value threshold of 0.05 corresponds to the value 1.3 in the vertical axes using the aforementioned transformation.

Each normalized dataset was subjected to statistical testing separately and the results were combined to form the final differential expression gene lists. The two methods had ~60% overlap. In order to identify significant alterations among all time points, 1-way ANOVA was applied to expression fold changes between expression in mastic oil and vehicle treated samples (p < 0.05, FDR<0.05) coupled with further filtering on fold change (>|0.2| in at least one time point in log_2 _scale). Statistical analysis coupled with fold change filtering yielded a list of 925 significantly differentiated genes (Additional file 1) which are depicted per time point using a volcano plot representation (Figure 1). In this figure, both statistical and empirical (fold change) thresholds are illustrated whereas red dots represent up-regulated genes and green dots represent down-regulated genes. Specifically, following the aforementioned criteria, the numbers of differentiated genes for each of the 3, 6, 12, 24 and 48 h time points were 519, 527, 503, 433 and 550 respectively.

### GOT-based meta-analysis

To gain evidence for possible altered biological processes, the list of genes resulting from ANOVA testing, was further subjected to ontological analysis based on Gene Ontology Terms (GOTs). Table 1 summarizes the over-represented GOTs with hypergeometric test p-value <0.01. GO categories of "Molecular Function" (MF) and "Biological Process" (BP) are shown, while GO categories representing very general functions, like "catalytic activity" or "hydrolase activity", are excluded. In order to further assess the correlation of the qualified GOTs, with specific biological processes, which can be conceptually visualized as a cohesive net of connected GOTs, a resampling algorithm was applied, as described in Methods section. The results (bootstrap p-values p_MF _= 0 and p_BP _= 0.001) support strongly the validity of the over-represented GO terms in Table 1 network of connections. In this sense, the derived GOTs strongly support the reliability of the mild fold change expression thresholds adopted in this study for the derivation of the significant gene list.

**Figure 2:**
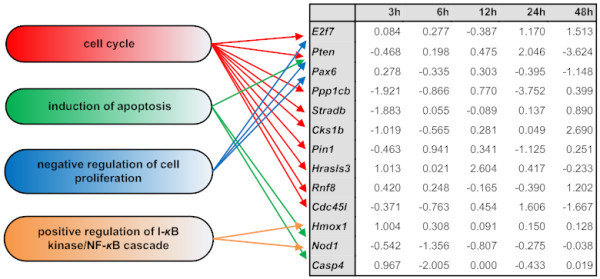
**Selected GO categories and relevant genes**. GO categories of interest and corresponding genes selected by fold change criteria as described in text. Values in 3, 6, 12, 24 and 48 h depict fold changes between mastic oil treatment and its corresponding control in log_2 _scale for each time point. The arrows from each GO category on the left to the genes in the table on the right show which genes are functionally connected to each GO category within the GO hierarchical model.

From the GO categories presented in Table 1, emphasis was given in four categories, namely "negative regulation of cell proliferation", "cell cycle", "induction of apoptosis"and "positive regulation of I-*κ*B kinase/NF-*κ*B cascade". Besides their established key role in cancer progression, the above categories were chosen on the basis of previous experimental evidence supporting that mastic oil inhibits LLC tumor growth in part through modulation of processes relevant to cell proliferation, apoptosis and inflammation [17]. Figure 2 presents the selected GO categories with the relevant genes and their fold change ratios. Each GO category is linked to the related genes with arrows starting from the GO categories. From the sets of genes belonging to these categories, only those presenting fold change above |1| in log_2 _scale in at least one time point were retained. Noteworthy, *Pten *(phosphatase and tensin homolog deleted on chromosome ten), is involved in three of the four GO categories, while in addition, there is evidence supporting a functional cross-talk between PTEN and NF-*κ*B signaling [29,30]. Therefore the observed modulation of *Pten *seemed to play a prominent role in mastic oil-mediated anti-tumor actions.

### RT-PCR validation and correlation with microarray data

**Figure 3:**
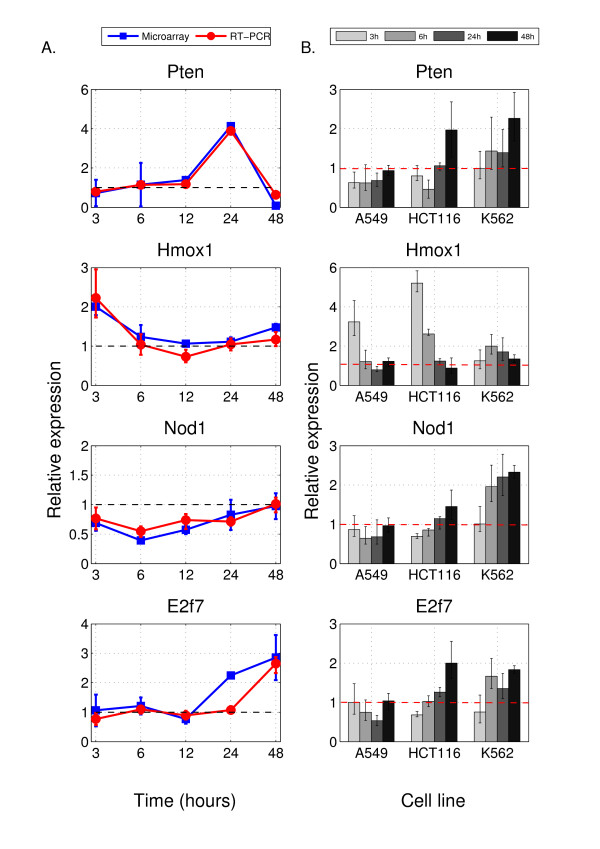
**Validation of microarray data by RT-PCR**. Four selected genes (*Pten*, *Hmox1*, *Nod1 *and *E2f7*) were analyzed by quantitative RT-PCR for microarray data confirmation. Error bars on each time point represent minimum and maximum relative expression values for RT-PCR data and standard deviations for the microarray experiment data. The dashed horizontal line across the value 1 on the vertical axes indicates that there is no difference in fold change ratios between treated and control cells. **A**. Expression levels measured by the two methods are shown for each time point. Specifically, for each of the depicted genes, the expression (in natural scale) of mastic oil treatment relative to its corresponding control, as derived from the microarray experiment and RT-PCR for each time point (3-48 h) is plotted and correlated. Overall, there is good correlation between microarray and RT-PCR validation data. **B**. The four selected genes from the analysis of microarray experiment were analyzed by quantitative RT-PCR in 3 cancer cell lines of human origin, namely A549, HCT116 and K562. Expression levels were measured for 4 distinct time points. Each panel shows the expression of mastic oil treatment relative to its control for each time point and for each cell line.

The results of microarray data analysis were further validated by quantitative RT-PCR analysis. Figure 3A presents mastic oil-induced changes in the expression of four representative genes picked from the list presented in Figure 2, namely *Pten*, *E2f7*, *Nod1 *and *Hmox1*, in all time points as assessed by both microarray and RT-PCR methods. Generally, there was a very good correlation in the overall profile of differential expression as well as in particular time points between the two methods.

Specifically, *Pten *shows constant expression at early time points (3-12 h) followed by significant up-regulation at 24 h and late modest down-regulation at 48 h as indicated by both microarray and RT-PCR data. *Pten *is the most elevated gene at 24 h and shows the highest agreement between microarray and RT-PCR data. *Hmox1 *is early induced at 3 h followed by almost constant expression in the remaining time points whereas RT-PCR and microarray data correlate very well. *Nod1 *is constantly repressed in all time points apart from 48 h, presenting high accordance between RT-PCR and microarray expression. Finally, *E2f7 *is constantly expressed between 3-12 h and late up-regulated at 24-48 h of mastic oil treatment. In the case of *E2f7 *although a discrepancy between microarray data and RT-PCR validation is observed at 24 h concerning the induction of the gene, yet the overall expression pattern remains similar across all time points.

Next, to investigate whether the aforementioned alterations are specific for mouse LLC cells or imply a more general response of cancer cells to mastic oil treatment, we examined by RT-PCR the expression profile of the above four selected genes in different types of human cancer cells. The results are presented in Figure 3B. Specifically, three different cell lines, namely lung adenocarcinoma (A549), colon carcinoma (HCT116) and erythromyeloblastoid leukaemia (K562) were used. A549 was selected as a human model of lung adenocarcinoma in analogy with LLC cells (lung adenocarcinoma in mice). HCT116 and K562 were used because of known susceptibility to mastic oil [13,16]. Gene expression was analyzed in four different time points (two early: 3 and 6 h, two late: 24 and 48 h) of mastic oil treatment. In agreement with the observed induction of *Pten *in LLC cells, we observe an up-regulation in both K562 and HCT116 cells, with a major induction at 48 h, but no response in A549 cells. *Hmox1 *demonstrates an increased expression in all of the three cell lines, mainly at early time points, which is in agreement with its induction at 3 h in the case of LLC cells. Regarding *Nod1*, in contrast to its down-regulation in LLC cells, we observe an elevation of its expression in K562 cells that was more modest in HCT116 cells and no significant response in A549 cells. Finally, *E2f7 *displays an induced expression both in K562 and HCT116 cells, in line with its profile in LLC cells but it is not significantly modified in A549 cells.

### PTEN mediates tumor cell growth inhibition by mastic oil

**Figure 4:**
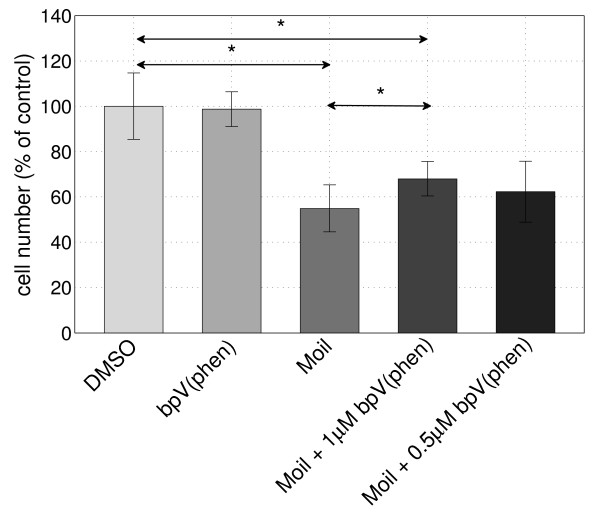
**Inhibition of PTEN reverses mastic oil effect on K562 cell growth**. K562 cells were treated with mastic oil alone (Moil, 0.007% v/v) or in the presence of PTEN inhibitor bpV(phen) (0.5 μM and 1 μM), bpV(phen) alone (1 μM) or DMSO as control. Cell numbers were measured after 24 h and results are expressed as mean percentage of DMSO control ± SD. The mean percentage of viable cells is significantly different between categories indicated by an asterisk as assessed by t-test (p < 0.05). The above statistics are derived from at least 3 independent experiments.

Based on the reported role of PTEN as a key negative regulator of the PI3K-AKT survival pathway [31] and given its observed up-regulation by mastic oil in three different tumor cell lines, we investigated whether the inhibitory effects of mastic oil on tumor cell growth are mediated by PTEN. For this purpose, dividing K562 cells were treated for 24 h with 0.01% mastic oil in the presence or not of 0.5-1 μM bpV(phen), a specific PTEN inhibitor [18], and the number of viable cells was determined. Mastic oil induced a decrease in cell number (viable cells were 54.9% of control treatment), which was partially reversed in the presence of bpV(phen) in a concentration-dependent manner (Figure 4). Specifically, in the presence of 1 μM and 0.5 μM of bpV(phen) the relevant cell number was 67.9% and 62.2% of control treatment, respectively.

### Dynamic clustering profile of gene expression data

**Figure 5:**
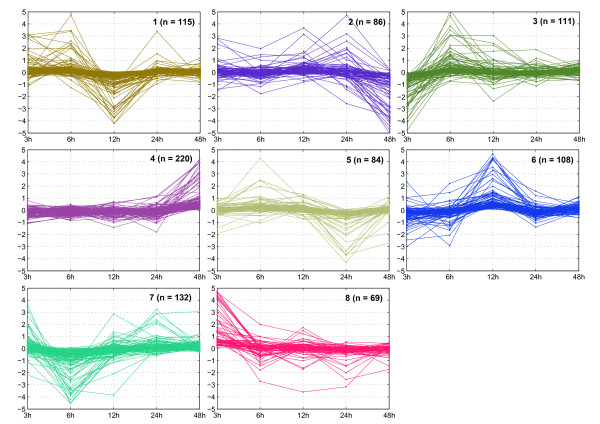
**Clustering of gene expression profiles**. Eight clusters of Illumina probe sets comprising similar expression profiles were identified by k-means clustering as described in methods. Each panel depicts one cluster of genes showing similar expression profile. The vertical axes depict fold changes in log_2 _scale between mastic oil treatment and its corresponding control for each time point, as derived from microarray data analysis. *n *depicts the number of probe sets in each cluster.

In order to identify groups of genes presenting similar expression profiles during the 3-48 h of mastic oil treatment and possibly comprising regulated "waves" of transcription, the list of 925 significantly differentiated genes was subjected to k-means clustering using Pearson correlation distance. Eight clusters containing genes with similar expression profiles were identified (Figure 5). Cluster-based GOT analysis connected regulated genes in each cluster to several functional categories where regulated genes are overrepresented. Due to the hierarchical structure of GO categories, the same gene can be linked to multiple GO terms.

Cluster 1 contains genes among which a number showing early up-regulation at 6 h followed by down-regulation at 12 h. Genes under cluster 1 are functionally connected to glutathione transferases activity as well as glutathione metabolism (*Gstt1*, *Gstz1*) which are down-regulated at 12 h. Glutathione comprises a well known antioxidant involved also in xenobiotic metabolism, the depletion of which has been reported to impair cell proliferation and cell growth and is an early event during apoptosis [32]. Cluster 2 contains genes, among which certain are constantly expressed at early time points and the majority of which manifests up-regulation in 24 h and late down-regulation at 48 h. Functions represented by genes in cluster 2 include cholesterol metabolic process (*Hdlbp*), negative regulation of cell proliferation (*Pten*, *Cebpa*) and fatty acid biosynthetic process (*Ndufab1*). Interestingly, *Pten *has been reported as a tumor-suppressor with role in cancer expansion and metastasis [33,34].

Cluster 3 depicts genes showing early down-regulation at 3 h followed by up-regulation at 6 h and then remaining mostly constant. Biological functions in cluster 3 include chemotaxis and chemokine activity (*Cxcl2*) as well as regulation of transcription (*Ncoa2*, *E2f2*). On the other hand, cluster 4 includes genes showing down-regulation at 24 h, followed by late induction at 48 h. Cluster 4 is functionally connected to cell cycle (*E2f7*, *Rnf8*, *Cks1b*, *Cdc45l*), cell division (*Cks1b*, *Mad1l1*), cell cycle arrest (*Bard1*, *Cdkn1a*) and negative regulation of cell proliferation (*E2f7*, *Cdkn1a*). Cluster 5 contains genes which are mostly up-regulated at 6 h and down-regulated at 24 h and is functionally connected with G1/S transition of mitotic cell cycle (*Camk2d*) and cell death (*Clu*).

Cluster 6, mainly comprises genes showing early down-regulation at 6 h followed by up-regulation at 12 h. Biological functions in cluster 6 include negative regulation of transcription (*Dedd2*, *Nr1h3*), cell growth (*Xrn2*) and activation of MAPK activity (*Map3k5*). Cluster 7 includes genes, the regulation of which is early-repressed at 6 h. A certain subgroup is up-regulated at 24 h. Cluster 7 is functionally connected to GTPase activity (*Gnaq*, *Rab5a*), positive regulation of I-*κ*B kinase/NF-*κ*B cascade (*Ppp5c*, *Nod1*), skeletal development (*Gnaq*) and response to hypoxia (*Ubqln1*). *Nod1 *is a known NF-*κ*B mediated pro-inflammatory agent involved in pro-apoptotic and anti-inflammatory signals [35]. Finally, cluster 8 groups together early up-regulated genes at 3 h and includes over-represented functions related to cell migration (*Nck2*), cell motility (*Mospd2*) and regulation of cell shape (*Centd3*). Generally, in all clusters the observed alterations in gene expression seem to be transient, as most genes exhibit altered expression for one or two time points and then their expression returns to control levels.

### Promoter analysis of genes in cluster 2

In order to investigate whether there is a common regulatory transcriptional mechanism underlying the common expression profiles revealed by clustering, we performed a representative promoter analysis in genes of cluster 2. This cluster contains 86 probe sets corresponding to 78 genes and includes *Pten*. Cluster 2 genes present a common expression pattern characterized by up-regulation in 24 h followed by down-regulation in 48 h. In order to find common putative transcription factor (TF) binding sites in at least a subset of cluster 2 genes, proximal promoter sequences from both mouse and human genomes were extracted from available databases and analyzed as described in Methods. Only common TF binding sites among the two species were considered. Table 2 summarizes statistically significant TF motif families common in promoter sequences of cluster 2 genes, sorted in descending order in terms of statistical significance.

**Table 2:** Common TF motif families in cluster 2

Family	p-value	% TF site containing promoters
V$CTCF	2.0652 × 10^-8^	80%
V$EGRF	2.0837 × 10^-8^	85%
V$ZBPF	3.3390 × 10^-8^	80%
V$AHRR	8.5552 × 10^-8^	69%
V$MAZF	2.0443 × 10^-6^	73%
V$SP1F	7.3263 × 10^-6^	85%
V$E2FF	1.6909 × 10^-4^	80%
V$EBOX	3.7640 × 10^-3^	69%

Common statistically significant TF motif families found in proximal promoter sequences of genes contained in cluster 2, which are present in both mouse and human genomes. The column family refers to families of functionally similar motifs grouped together. The p-value is the probability to obtain a greater or equal number of sequences with a match in a random sample of the same size as the input sequence set. The percentage column depicts the percentage of genes in cluster 2 whose promoters have at least one match with the respective motif family. In each family identifier, V$ stands for "Vertebrates". Percentages and p-value calculations are based on mouse promoters.

## Discussion

Lately, there is an increasing interest for exploring the tumor preventive actions of mastic gum and its essential oil, a natural blend of bioactive terpenes. Results obtained to date support that mastic gum/oil can inhibit the growth of several cancer types *in vitro *[13-16]. In addition, the anti-tumor potential of mastic oil has been recently confirmed *in vivo *on the experimental growth of Lewis lung adenocarcinoma (LLC) tumors, an aggressive type of mouse lung cancer and some of the underlying mechanisms have been investigated [17]. In the present study, through a genome-wide transcriptomic approach we identified for the first time the dynamic alterations occurring in gene expression of LLC cells in response to mastic oil treatment at different incubation periods (3, 6, 12, 24 and 48 h) and undertook a detailed analysis of the involved mechanisms using bioinformatic methodologies. Furthermore, we followed by RT-PCR the expression of four selected genes, namely *Pten*, *Hmox1*, *E2f7 *and *Nod1 *in LLC as well as in other cancer cell lines of human origin, in order to reveal potential common patterns of response to mastic oil among different tumor types.

Microarray data normalization by two different algorithms provided a list of 925 genes with a significantly modified expression in mastic oil treated cells compared to untreated controls. RT-PCR selectively applied on the above mentioned genes due to their particular interest, further confirmed the expression profiles found by the microarray approach. Meta-analysis performed on the complete set of differentially expressed genes using the GO database (Table 1), comprised a statistically strong cohesive net of functions as derived from the GO tree, thus further supporting the reliability of the adopted fold change expression thresholds. This analysis demonstrated that mastic oil influences significantly a number of fundamental cellular processes such as those related to cell cycle, apoptosis, signal transduction and regulation of transcription. Also some more specific functions (e.g. glutathione transferase activity and metabolism), linked to detoxification of endogenous or exogenous metabolites, a well-known mechanism of cancer chemoprevention by several phytochemicals, including terpenoids [36-38], are affected.

It is worth mentioning that alterations in GO categories related to "negative regulation of cell proliferation", "cell cycle", "positive regulation of I-*κ*B kinase/NF-*κ*B cascade" and "induction of apoptosis" correlated very well with existing experimental evidence supporting that mastic oil causes regression of tumor growth by inhibiting cancer cell proliferation and survival [13,16,17], reducing tumor-associated expression of inflammatory mediators [17] and down-regulating NF-*κ*B transcriptional activity [14,17].

An interesting result of our analysis was that among the targeted by mastic oil genes corresponding to the GO categories presented in Figure 2, *Pten *appeared to be relevant to three of them. Its expression was further assessed by RT-PCR and found to be late up-regulated in LLC, HCT116 and K562 cells. This gene which encodes for PTEN, a dual-specificity phosphatase, has lately gained much attention as a tumor-suppressor due to its prominent inhibitory role in cancer expansion and metastasis [33,34]. Although little is known about the upstream regulatory pathways for PTEN, one of its most recognized down-stream actions is the blockade of PI3K/AKT signaling through de-phosphorylation of phosphatidylinositol (3,4,5) tri-phosphate (PIP3) which is generated by PI3K to mediate AKT activation [31,36]. Active AKT then phosphorylates a plethora of targets to activate cell cycle, prevent apoptosis and trigger NF-*κ*B signaling [37]. Furthermore PTEN has been shown to antagonize tumor necrosis factor (TNF)-stimulated NF-*κ*B-dependent gene expression, thus sensitizing cells to TNF-induced apoptosis [29]. Thereby the induction of *Pten *expression by mastic oil may be considered as an essential upstream effect accounting for the anti-proliferative, pro-apoptotic and anti-inflammatory actions of mastic oil through blockade of AKT and NF-*κ*B transcriptional activity. In line with this, we found that pharmacological blockade of PTEN by bpV(phen) partially reversed the anti-tumor growth effects of mastic oil in K562 cells. These results indicate the active involvement of PTEN but also of other target pathways in tumor growth suppression by mastic oil. Compatible to this hypothesis, perillyl alcohol (one of the best studied mastic oil components) and mastic gum have been shown able to reduce the levels of phosphorylated (active) AKT [14,38] and in addition mastic oil to down-regulate the constitutive and TNF-α-induced NF-*κ*B-dependent gene transcription [17].

In conformity with a negative regulation of cell proliferation and/or survival by mastic oil was also the observed up-regulation of two other genes, namely *E2f7 *and *Hrasls3*. The expression of *E2f7 *was also determined by RT-PCR and found to be induced in LLC, HCT116 and K562 cells. E2F7 is a transcription factor which, in contrast to the other members of E2F family, has been shown to act as a repressor of several genes known to promote tumor cell proliferation [39]. Concerning *Hrasls3*, a member of the HREV107 type II tumor suppressors, it has been shown to exert anti-proliferative and pro-apoptotic actions and to be down-regulated by Ras-dependent signaling [40-42]. In correlation, mastic oil has been shown able to reduce Ras levels in LLC cells in vivo and *in vitro *[17], probably as an indirect consequence of mevalonate pathway blockade by its isoprenoid components [8], leading to disruption of Ras signaling. Furthermore, although *Nod1 *was found to be repressed in LLC, it was elevated in HCT116 and K562 cells indicating an heterogeneity in response to mastic oil.

Moreover, transient up-regulation of *Hmox1 *in all tested cell lines corroborates with the inhibitory effects of mastic oil on tumor-related inflammatory response [17]. This gene has been reported to be highly inducible by a variety of stress stimuli (hypoxia, inflammation, heavy metals, UV radiations) including several phytochemicals [43]. HMOX1, a heme-degrading enzyme, has been shown to exert anti-oxidative as well as a broad range of anti-inflammatory activities attributed to inhibition of pro-inflammatory mediators and negative regulation of NF-*κ*B signaling [43,44]. Besides, HMOX1 is known to be involved in the process of xenobiotic metabolism by inactivating chemically reactive metabolites and scavenging reactive oxygen species [45]. This finding along with the proposed by GO analysis modification of glutathione transferase activity (Table 1) support that activation of detoxifying enzymes might also contribute to cancer chemoprevention by mastic oil in agreement with previous studies indicating the anti-oxidative potential of mastic oil [11,46]. In contrast, Doi *et al *[47], using a medium-term carcinogenesis assay in rat liver, have shown that *Hmox1 *was one of the most repressed genes after treatment with mastic oil. In our system however over-expression of *Hmox1 *was observed as an early response to mastic oil treatment, while in [47] gene expression profile was determined after administration of mastic oil for 6 weeks. In addition, Doi *et al *reported the formation of prenoeoplastic lesions in rat liver after treatment with mastic oil which is not in accordance with our results. It is possible that the unique features of each experimental system, the intrinsic differences of each cell type and/or the presence of some distinct components in mastic gum compared to mastic oil, account for such contradictory effects. For instance Paraschos *et al *[48] recently reported that a polymer present in mastic gum could hinder its anti-bacterial activity and reduce the bioavailability of bioactive components. In any case, those controversial results support the need of further studies in order to investigate mastic oil metabolism and bioavailability and specify safe dosage limits.

Finally, by applying k-means clustering the complete set of genes was organized in eight clusters each representing a specific gene expression profile, probably indicating a common regulatory transcriptional mechanism (Figure 5). To test this hypothesis, we performed a representative promoter analysis looking for putative common *cis*-elements in genes grouped in cluster 2 (Table 2). Putative elements belonging to the EGR family were identified in 85% of the promoters of cluster 2 genes. Functional EGR binding sites have been identified in the promoters of several tumor suppressor genes, including *Pten *[49,50]. In particular, EGR1 has been shown to directly transactivate *Pten*. Furthermore, elements of the AHRR family, containing the Xenobiotic Response Element (XRE) and closely related TF binding sites involved in transcriptional stimulation of several xenobiotic metabolizing enzymes by several phytochemicals [51,52], were identified in 69% of cluster 2 genes.

## Conclusions

**Figure 6:**
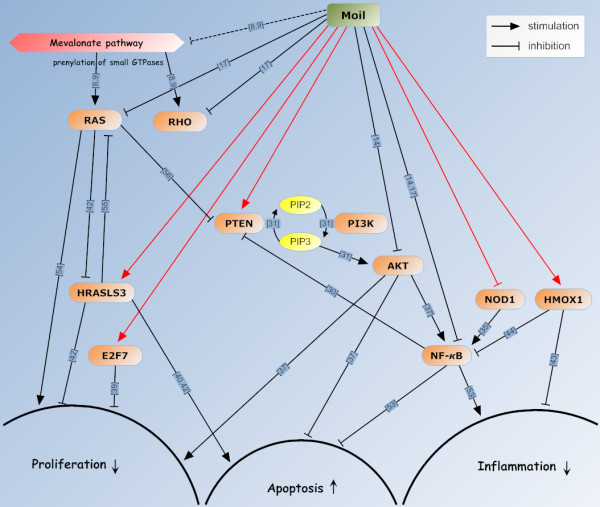
**Model of mastic oil action mechanisms in LLC cells**. Schematic representation of target molecules and pathways underlying mastic oil-mediated chemopreventive effects on tumor cell proliferation, apoptosis and inflammation based on available literature (black arrows) and evidence as derived from the present study for LLC cells (red arrows). Dashed line indicates prediction of activity based on experimental evidence from mastic oil components. Numbers on arrows correspond to respective references indicated in the text and also interactions described in references [53-56].

Overall, this study, by combining microarray gene expression profiling with bioinformatic analyses on a model of mouse lung adenocarcinoma provided novel evidence for the target molecules and pathways underlying mastic oil inhibitory actions on tumor cell growth and survival. Figure 6 shows a proposed mechanism of action including several key molecules involved in cell proliferation, apoptosis and inflammation based on our microarray study on mastic oil treated LLC cells and the relevant literature.

## Abbreviations

AHRR: aryl-hydrocarbon receptor related factors; bpV(phen): potassium bisperoxo (1,10-phenanthroline) oxovanadate; Casp4: caspase 4; Cdc45l: cell division cycle 45-like; Cks1b: CDC28 protein kinase 1b; CTCF: CCCTC-binding factor; EGRF: early growth response related factors; FDR: False Discovery Rate; GO: Gene Ontology; Hmox1: heme oxygenase 1; Hrasls3: Hras-like suppressor 3; MAZF: MYC-associated zinc fingers; NF-κB: nuclear factor kappa beta; Nod1: nucleotide-binding oligomerization domain containing 1; Pax6: paired box gene 6; Pin1: protein (peptidyl-prolyl cis/trans isomerase) NIMA-interacting 1; Ppp1cb: protein phosphatase 1, catalytic subunit, beta isoform; Pten: phosphatase and tensin homolog deleted on chromosome ten; Rnf8: ring finger protein 8; SP1F: Specificity protein 1 related factors; Stradb: STE20-related kinase adaptor beta; TF: Transcription factor; ZBPF: zing binding protein factors. Throughout the text, gene names are written using *Sentence case italic *letters and protein names are written using CAPITAL letters.

## Competing interests

The authors declare that they have no competing interests.

## Authors' contributions

PM contributed to computational analyses and meta-analyses and draft writing. OP performed the biological experiments, computational promoter analysis and draft writing. AC participated in the supervision of the computational analyses and meta-analyses. HL contributed to the conception and design of the study, supervision of biological experiments, data interpretation and manuscript writing. CR participated in the design of the study and manuscript revision. FNK participated in the study design and supervised the overall process. All authors have read and approved the final manuscript.

## Pre-publication history

The pre-publication history for this paper can be accessed here:

http://www.biomedcentral.com/1755-8794/2/68/prepub

## Supplementary Material

Additional file 1 **List of 925 differentially expressed genes in response to mastic oil**. The list of 925 differentially expressed genes and their cluster memberships as derived after statistical and clustering analysis as described in Methods. Several annotation elements such as GenBank accession numbers and RefSeq IDs are included together with fold change ratios for each differentially expressed gene. Click here for file
